# Wasted research when systematic reviews fail to provide a complete and up-to-date evidence synthesis: the example of lung cancer

**DOI:** 10.1186/s12916-016-0555-0

**Published:** 2016-01-20

**Authors:** Perrine Créquit, Ludovic Trinquart, Amélie Yavchitz, Philippe Ravaud

**Affiliations:** Centre de Recherche Epidémiologie et Statistique Sorbonne Paris Cité, INSERM U1153, Paris, France; Université Paris Descartes – Sorbonne Paris Cité, Paris, France; Assistance Publique-Hôpitaux de Paris, Hôpital Hôtel-Dieu, Centre d’Epidémiologie Clinique, Paris, France; Cochrane France, Paris, France; Department of Epidemiology, Mailman School of Public Health, Columbia University, New York, USA

**Keywords:** Meta-analysis as topic, Systematic reviews, Randomized controlled trials, Network meta-analysis, Non-small cell lung cancer

## Abstract

**Background:**

Multiple treatments are frequently available for a given condition, and clinicians and patients need a comprehensive, up-to-date synthesis of evidence for all competing treatments. We aimed to quantify the waste of research related to the failure of systematic reviews to provide a complete and up-to-date evidence synthesis over time.

**Methods:**

We performed a series of systematic overviews and networks of randomized trials assessing the gap between evidence covered by systematic reviews and available trials of second-line treatments for advanced non-small cell lung cancer. We searched the Cochrane Database of Systematic Reviews, Database of Abstracts of Reviews of Effects, MEDLINE, EMBASE, and other resources sequentially by year from 2009 to March 2, 2015. We sequentially compared the amount of evidence missing from systematic reviews to the randomized evidence available for inclusion each year. We constructed cumulative networks of randomized evidence over time and evaluated the proportion of trials, patients, treatments, and treatment comparisons not covered by systematic reviews on December 31 each year from 2009 to 2015.

**Results:**

We identified 77 trials (28,636 patients) assessing 47 treatments with 54 comparisons and 29 systematic reviews (13 published after 2013). From 2009 to 2015, the evidence covered by existing systematic reviews was consistently incomplete: 45 % to 70 % of trials; 30 % to 58 % of patients; 40 % to 66 % of treatments; and 38 % to 71 % of comparisons were missing. In the cumulative networks of randomized evidence, 10 % to 17 % of treatment comparisons were partially covered by systematic reviews and 55 % to 85 % were partially or not covered.

**Conclusions:**

We illustrate how systematic reviews of a given condition provide a fragmented, out-of-date panorama of the evidence for all treatments. This waste of research might be reduced by the development of live cumulative network meta-analyses.

**Electronic supplementary material:**

The online version of this article (doi:10.1186/s12916-016-0555-0) contains supplementary material, which is available to authorized users.

## Background

For many conditions, multiple competing treatments are available, many of which have been assessed in randomized trials [1]. Clinicians and patients who are making medical decisions need to know which treatment works best among all treatments for the condition of interest. They increasingly turn to systematic reviews and meta-analyses for current evidence-based assessments of the relative benefits and harms of treatments.

To decide the best treatment for a patient, clinicians and patients need a comprehensive, up-to-date synthesis of evidence for all treatments available for a given condition [2–4]. This synthesis could be provided by considering the whole set of conventional meta-analyses on all treatment comparisons or a network meta-analysis [5].

However, systematic reviews as currently performed may fail to meet clinicians’ and patients’ needs [6]. Systematic reviews and meta-analyses are insufficiently informative if they do not cover all alternative treatments or do not include all available current evidence. In fact, most meta-analyses have a narrow scope and focus on specific treatments [7]. Moreover, many meta-analyses become quickly out-of-date because clinically important evidence can accumulate rapidly, but updating a systematic review can be as costly and time-consuming as the original review [8, 9]. This failure to rigorously synthesize the totality of relevant evidence may have a detrimental effect on treatment decisions and future research planning.

The exponential growth in publications of randomized trials, especially in oncology, increases clinicians’ and patients’ need for broad meta-analyses encompassing all the evidence for all competing treatments [10]. Lung cancer, in particular, remains the fifth leading cause of disability-adjusted life years in developed countries and represents a key area of current therapeutic innovation [11]. With recent progresses in therapeutics, the number of patients with advanced non-small cell lung cancer (NSCLC) who receive second-line treatments is increasing, but which second-line treatment to recommend is unclear.

We used the example of NSCLC to quantify the waste of research related to systematic reviews failing to provide a complete and up-to-date synthesis of evidence over time.

## Methods

We first used a comprehensive strategy to repeatedly identify all randomized trials, with published and unpublished results, and all systematic reviews of second-line treatments for advanced NSCLC available up to the end of each year from 2009 to 2015. Second, we sequentially assessed the amount of randomized evidence that was covered by systematic reviews collectively: for the years 2009 to 2015, we assessed the articles published up to December 31 of each of those years for proportion of treatments, treatment comparisons, trials, and patients covered by systematic reviews on this topic, with comparison to the total randomized evidence available at each time.

### Identification of randomized trials

#### Eligibility criteria

We considered randomized trials of second-line treatments compared to each other or against a placebo or best supportive care in patients with advanced (stage IIIB–IV) NSCLC. We considered any cytotoxic monochemotherapy, any targeted treatment, any combination of a cytotoxic monochemotherapy and targeted treatment, and any combination of two targeted treatments (complete list in Additional file 1: Appendix 1). We excluded trials assessing doublet chemotherapy and comparing two different administration schemes. We excluded trials focusing exclusively on patients with epidermal growth factor receptor (EGFR)-activating mutation or anaplastic lymphoma kinase (ALK) rearrangement, because it represented a specific minority sub-group of all advanced NSCLC.

#### Search strategy

We searched for reports of randomized trials in the Cochrane Central Register of Controlled Trials, MEDLINE, and EMBASE (search equations in Additional file 1: Appendix 2) with no restriction on language, status, or year of publication, and searched other resources [12]: 1) previous systematic reviews (see below); 2) reference lists of all selected trials; 3) conference abstracts (from the American Society of Clinical Oncology Meeting, European Society of Medical Oncology Congress, and World Lung Cancer Conference); 4) non-industry trial registries and results databases (ClinicalTrials.gov and EudraCT); 5) industry trial registries and results databases; and 6) regulatory agency online databases (US Food and Drug Administration and European Medicines Agency); details in Additional file 1: Appendix 3. We contacted trialists to request complete results for all trials identified as “completed” on ClinicalTrials.gov but without published results and for all trials with conference abstracts but no full-text articles. The last search was conducted on March 2, 2015.

### Identification of systematic reviews

#### Eligibility criteria

Systematic reviews of randomized trials of second-line treatments for advanced NSCLC were eligible. We selected reviews that addressed at least one comparison between the treatments considered previously, whether they included a meta-analysis or not. We excluded reviews that did not report clearly stated objectives, eligibility criteria for trials, or a systematic search strategy; reviews combining first- and second-line data were ineligible; reviews focusing exclusively on patients with EGFR mutation or ALK rearrangement or on doublet chemotherapy, or comparing two different administration schemes; and reviews that did not provide the list of included trials.

#### Search strategy

We searched the Cochrane Database of Systematic Reviews, the Database of Abstracts of Reviews of Effects, MEDLINE, EMBASE (search equations in Additional file 1: Appendix 4), and other resources: conference abstracts from the American Society of Clinical Oncology Meeting, European Society of Medical Oncology Congress, and World Lung Cancer Conference; and the PROSPERO international prospective register of systematic reviews for completed or published systematic reviews (details in Additional file 1: Appendix 5). There was no restriction on language, status, or year of publication. The last search was conducted on March 2, 2015.

### Selection of studies and extraction of data

Two authors independently and in duplicate examined titles, abstracts, and full-text articles to determine the eligibility of randomized trials and systematic reviews. We pilot-tested the eligibility criteria on a sample of 100 records (for the selection on titles and abstracts) and 10 reports (for the selection on full-text articles) to ensure that the selection criteria were applied consistently by the two authors. Disagreements were discussed with a third author. All data were independently extracted by two authors who used a standardized form.

For each trial, we extracted the dates of publication of the full-text article(s) (online publication, if any) and conference abstract(s), date of results posting on non-industry and industry trial registries, and date of publication of reports by regulatory agencies. We also extracted the treatments assessed, number of randomized patients in each arm, study phase (II or III), and reported outcomes (overall survival and progression-free survival).

For each systematic review, we extracted the list of relevant trials selected, the publication date (online publication, if any), date of last search of trials, number of trials included, and type of treatment compared. We assessed the funding source (industry, non-industry, no funding, or not reported), whether the review was an update of a previously published review, and whether a network meta-analysis was performed. Finally, we assessed the scope of each systematic review (i.e., interventions and comparators assessed). We identified whether the review focused on one specific treatment explicitly (alone or combined with other treatments), lumped different treatments of the same type together (e.g., monochemotherapy or targeted therapy), and lumped different types of treatments together (e.g., monochemotherapy and EGFR tyrosine kinase inhibitors considered the same intervention).

Two reviewers independently assessed the methodological quality of the systematic reviews, with a formal consensus process in case of disagreement. We used AMSTAR, a measurement tool created to assess the methodological quality of systematic reviews [13], which has been validated [14, 15]. We assessed the four items pertaining to duplicate study selection and data extraction, comprehensive literature search (at least two electronic sources and one supplementary strategy among reviews, experts, or reviewing the references), searching for reports regardless of their publication type, and providing a list of included and excluded trials. Searching for trials regardless of their publication type was judged inadequate when authors did not report searching the grey literature (conference abstracts, non-industry trial registries and results databases, industry trial registries and results databases, regulatory agency online databases) or excluding reports based on language. We focused on these four specific items because the methods used for the identification and selection of studies are directly related to a potential gap between the amount of randomized evidence covered by systematic reviews and the amount of randomized evidence available for inclusion, and other domains are unrelated.

### Definition of randomized evidence available for inclusion in systematic reviews

We pre-specified the year 2009 as a starting point for our analyses in order to allow for a sufficient amount of evidence (in terms of both randomized trials and available systematic reviews) regarding the comparison between competing second-line treatments for advanced NSCLC to initiate a comparison between the available randomized evidence and that covered by systematic reviews. From 2009 to 2015, we identified the cumulative list of trials eligible for inclusion in systematic reviews; we checked that each trial identified would have been eligible for inclusion in at least one systematic review (i.e., corresponded to the selection criteria in terms of patients, interventions, and comparators). For each trial, we identified the earliest report of results and considered the corresponding publication date as when the trial became eligible for inclusion in systematic reviews. Considering the inevitable time lag between completion and publication, most recently published trials could not be selected by any systematic review, so we considered a 6-month lag period as recommended by the Cochrane Collaboration (i.e., we listed all trial results published up to July 1 each year, and up to August 31, 2014 for 2015) [16]. We also compiled the cumulative list of treatments and treatment comparisons assessed in eligible trials; finally, we calculated the cumulative number of patients included in trials as a measure of the available amount of randomized evidence.

### Definition of randomized evidence covered by systematic reviews

We considered all systematic reviews published up to December 31 each year from 2009 to 2015 (up to March 2 for 2015). The reference date for a systematic review was the publication date of the full-text article or online publication date, if any. We compiled the cumulative list of all relevant trials selected by these systematic reviews and the cumulative list of treatments and treatment comparisons and cumulative number of included patients in the trials selected by the systematic reviews.

### Assessment of randomized evidence not covered by systematic reviews

We evaluated the overall number and proportion of treatments, treatment comparisons, trials, and patients not covered by systematic reviews from 2009 to 2015.

We constructed cumulative networks of randomized evidence. Each node was a treatment and each edge was a treatment comparison (i.e., an edge connected two nodes when at least one randomized trial compared the two corresponding treatments). In multi-arm trials, doses of the same drug were lumped under a common node. The node size was proportional to the total number of patients randomly allocated to the corresponding treatment across all randomized trials available for inclusion; we represented the proportions of randomized patients not actually covered by systematic reviews by pie charts overlaid on nodes in the network. The edge width was proportional to the total number of randomized trials between the corresponding treatments available for inclusion; we represented the proportions of trials not selected by systematic reviews by a percentage bar chart overlaid on edges in the network. The evidence for a treatment comparison was considered partially covered when systematic reviews did not cover all the evidence available for this treatment comparison.

In sensitivity analyses, we discarded trials potentially ineligible for inclusion in any systematic review: trials of drugs that did not successfully pass phase II; trials that did not report treatment effects on overall survival or progression-free survival; and trials with results reported in conference abstracts only. In a last sensitivity analysis, the lag period to define randomized evidence available for inclusion in systematic reviews was defined by the last date of search for the last published systematic review.

Analyses involved use of R version 3.2.1 (R Development Core Team, Vienna, Austria).

## Results

### Randomized trials of second-line treatments for NSCLC

**Fig. 1:**
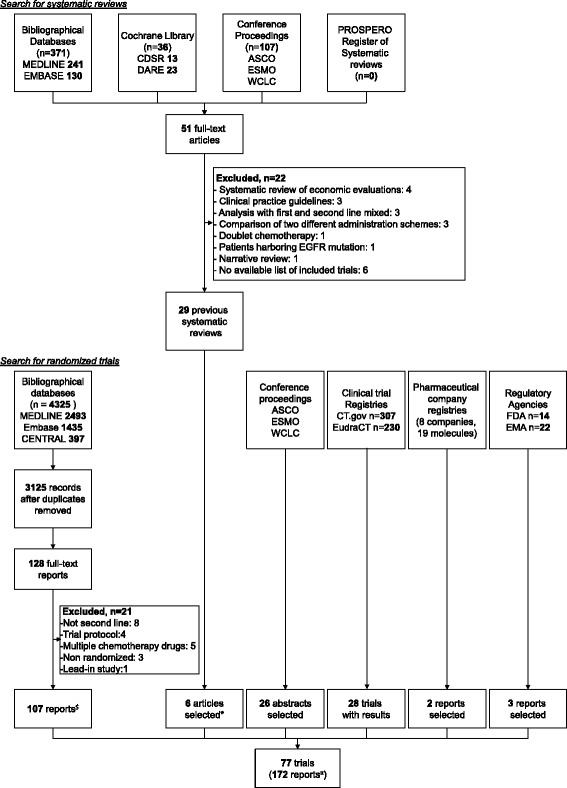
Flow diagram of selection of systematic reviews and randomized controlled trials of second-line treatments in advanced non-small cell lung cancer. ^*^Additional full-text articles not identified by searching bibliographical databases; ^$^63 full-text articles and 44 conference abstracts; ^¤^69 full-text articles, 70 conference abstracts, 28 posted results, and 5 industry/FDA reports

**Fig. 2:**
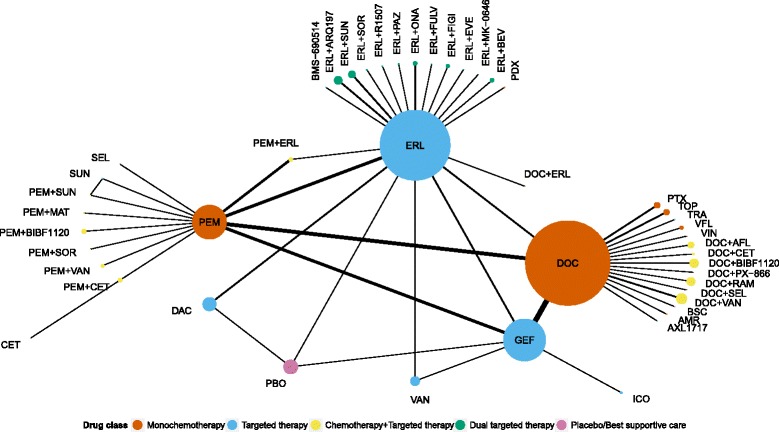
Network of 77 randomized controlled trials of second-line treatments in advanced non-small cell lung cancer. The thickness of connecting lines indicates the number of available comparisons. The size of each node is proportional to the number of patients allocated to the corresponding treatment. AFL: aflibercept; AMR: amrubicin; ARQ197: tivantinib; BEV: bevacizumab; BIBF1120: nintedanib; BSC: best supportive care; CET: cetuximab; DAC: dacomitinib; DOC: docetaxel; ERL: erlotinib; EVE: everolimus; FIGI: figitumumab; FULV: fulvestrant; GEF: gefitinib; ICO: icotinib; MAT: matuzumab; MK-0646: dalotuzumab; ONA: onartuzumab; PAZ: pazopanib; PBO: placebo; PDX: pralatrexate; PEM: pemetrexed; PTX: paclitaxel; RAM: ramucirumab; SEL: selumetinib; SOR: sorafenib; SUN: sunitinib; TOP: topotecan; TRA: trametinib; VAN: vandetanib; VFL: vinflunine; VIN: vinorelbine

We identified 77 eligible trials with results available between May 2000 and November 2014 (Fig. 1). The results of 62 trials were published in 69 articles; among these 62 trials, 43 had results reported in other sources (16 in conference abstracts, 10 in non-industry trial registries and results databases or regulatory agency online databases, and 17 in both). The remaining 15 trials (20 %) had unpublished results (12 conference abstracts, 2 non-industry trial registries and results databases or regulatory agency online databases, and 1 both). In all, 61 trials (79 %) were registered at ClinicalTrials.gov. The 77 trials included 28,636 patients (median [Q1–Q3] 168 [100–559] patients); 72 included two arms, two compared three different treatments, and three compared one drug to two different doses of another drug, which were lumped together. Fig. 2 shows the complete network of evidence, with 45 different treatments and 54 treatment comparisons against each other or best supportive care or placebo. With 47 nodes in the network, there were 47 × 46/2 = 1,081 possible pairwise comparisons. The available direct evidence informs 54 comparisons (5 %).

### Systematic reviews of second-line treatments for NSCLC

We identified 29 systematic reviews published from April 2001 to February 2015 (Table 1) [17–45]. The first published review was a Cochrane review and all subsequent reviews were published in journal venues. The number of reviews doubled in 2014, from 16 to 27 (Additional file 1: Appendix 6). The median time between the last review search and publication was 9 months [Q1–Q3 5–13 months]. The industry was involved in 10 % of the reviews and not involved in 41 %, and the funding source was unclear in 41 %. The reviews addressed 19 comparisons, with 26 reviews lumping different treatments or different types of treatments together, considering them as the same intervention or comparator. Six reviews focused on one specific treatment of main interest. Two reviews performed network meta-analyses but did not cover all available treatments. Only one of the 29 reviews was an update of a previously published review.

**Table 1:** Characteristics of 29 selected systematic reviews

Systematic review	Last search	Publication date	Number of trials	Funding source	Intervention	Comparator	Specific treatment	Different treatments lumped together	Different types of treatments lumped together
Bonfill 2002	Jul 2001	Apr 2001	1	Non-industry	CTx	PBO or BSC	No	Yes	No
Tassinari 2009	Jul 2008	Feb 2009	3	NR	CTx or EGFRTKI	BSC	No	Yes	Yes
Yang 2014	Dec 2013	May 2014	2	NR	EGFRTKI	PBO	No	Yes	No
Wong 2013^‡^	SMay 2012	Oct 2013	4	NR	EGFRTKI	CTx or PBO	No	Yes	Yes
Barlesi 2006	Feb 2005	Dec 2005	4	NR	DOC	CTx or BSC	No	Yes	Yes
Al-Saleh 2012	Jan 2010	Feb 2012	1	Industry	PEM	CTx	Yes	Yes	No
Perez-Moreno 2014	Apr 2012	Mar 2014	1	Non-industry	PEM	CTx	Yes	Yes	No
Jiang 2011	Feb 2010	Dec 2010	4	Non-industry	GEF	DOC	Yes	No	No
Qi 2012c	Mar 2012	Oct 2012	8	Non-industry	EGFRTKI	CTx	No	Yes	No
Gao 2013^‡^	NR	Jun 2013	3	NR	EGFRTKI	CTx	No	Yes	No
Lee 2014	Dec 2013	Apr 2014	7	Non-industry	EGFRTKI	CTx	No	Yes	No
Zhao 2014	Jul 2013	Apr 2014	6	Non-industry	EGFRTKI	CTx	No	Yes	No
Li 2014b	Jul 2013	Jul 2014	10	None	EGFRTKI	CTx	No	Yes	No
Vale 2014	Jan 2014	Nov 2014	14	Non-industry	EGFRTKI	CTx	No	Yes	No
Qi 2012a	Mar 2011	May 2011	8	NR	DOC + (CTx or TT)	DOC	No	Yes	Yes
Jin 2014^*^	Dec 2013	Sep 2014	12	Non-industry	DOC + (CTx or TT)	DOC	No	Yes	Yes
Qi 2012b	May 2011	Jan 2012	5	NR	PEM + (CTx or TT)	PEM	No	Yes	Yes
Sun 2014	Feb 2012	Apr 2014	4	NR	PEM + (CTx or TT)	PEM	No	Yes	Yes
Qi 2011	Jul 2011	Oct 2011	4	NR	CTx + VAN or VAN	CTx or EGFRTKI	Yes	Yes	Yes
Tao 2012	Sep 2011	Mar 2012	5	NR	CTx + VAN or VAN	CTx or EGFRTKI	Yes	Yes	Yes
Tassinari 2012	Jun 2010	Dec 2012	4	NR	DOC	CTx or EGFRTKI	No	Yes	Yes
Qi 2013	May 2012	Feb 2013	8	Non-industry	ERL + TT	ERL	No	Yes	No
Cui 2013	Dec 2011	Apr 2013	8	Non-industry	BEV + (CTx or EGFRTKI)	CTx or EGFRTKI	Yes	Yes	Yes
					EGFRTKI	CTx or PBO	No	Yes	Yes
Li 2014a	Dec 2013	Apr 2014	14	None	CTx + TT	CTx	No	Yes	No
Liang 2014	Jan 2014	Oct 2014	10	Non-industry	MATKI + (CTx or EGFRTKI) or MATKI	CTx or EGFRTKI or PBO	No	Yes	Yes
Sun 2015	Oct 2014	Jan 2015	2	NR	BEV + EGFRTKI	EGFRTKI	No	Yes	No
Xiao 2015	Sep 2014	Feb 2015	5	Non-industry	CTx + EGFRTKI	CTx or EGFRTKI	No	Yes	Yes
Hawkins 2009^†^	Oct 2007	Apr 2009	6	Industry	DOC vs PEM vs ERL vs GEF		No	No	No
Popat 2015^†^	Mar 2014	Dec 2014	9	Industry	CTx vs TT vs CTx + TT vs (PBO or BSC)		No	No	No

^*^Update of Qi 2012a; ^†^network meta-analysis; ^‡^conference abstracts. BEV: bevacizumab; BSC: best supportive care; CTx: monochemotherapy; DOC: docetaxel; EGFRTKI: EGFR tyrosine kinase inhibitors; ERL: erlotinib; GEF: gefitinib; MATKI: multi-targeted antiangiogenic tyrosine kinase inhibitors; NR: not reported; PBO: placebo; PEM: pemetrexed; TT: targeted therapy; VAN: vandetanib

Regarding the methodological quality of the 29 systematic reviews, 45 % of reviews lacked information on independent study selection and data extraction, 31 % a comprehensive literature search, and 45 % a search for reports regardless of their publication type. Of note, 17 % of reviews lacked information on a search for conference abstracts, 86 % a search for non-industry trial registries and results databases, 97 % a search for industry trial registries and results databases, and 97 % a search of regulatory agency online databases. In all, 79 % of systematic reviews did not report duplicate study selection and data extraction, comprehensive literature search, and searching for reports regardless of their publication type. Finally, 7 % of reviews provided a list of included and excluded trials.

### Randomized evidence not covered or partially covered by systematic reviews

**Fig. 3:**
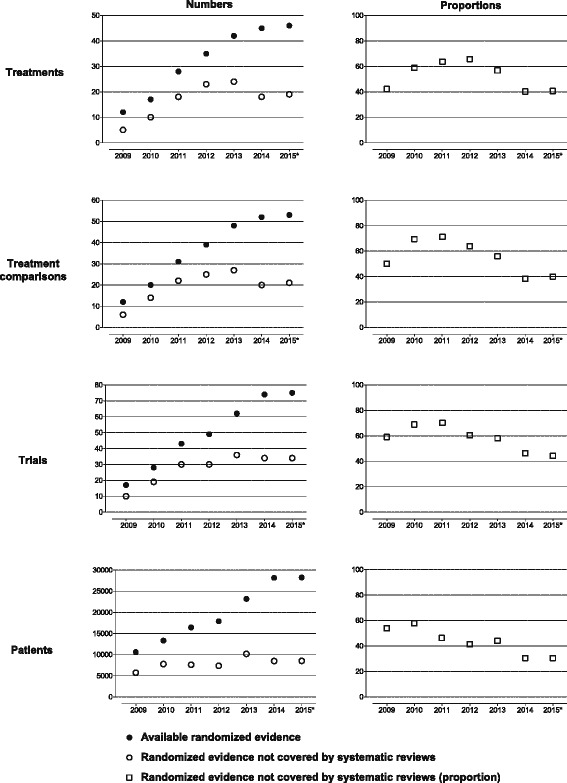
Amount of treatments, treatment comparisons, trials, and patients not covered by systematic reviews from 2009 to 2015. ^*^The last search for randomized trials and systematic reviews was conducted on March 2, 2015

From 2009 to 2015, the amount of randomized evidence covered by existing systematic reviews was consistently incomplete: 40 % to 66 % of treatments; 38 % to 71 % of treatment comparisons; 45 % to 70 % of trials; and 30 % to 58 % of patients were missing (Fig. 3). In 2014, 27 reviews still did not cover 18 treatments (40 %), 20 treatment comparisons (38 %), 34 trials (46 %), and 8,486 patients (30 %).

**Fig. 4:**
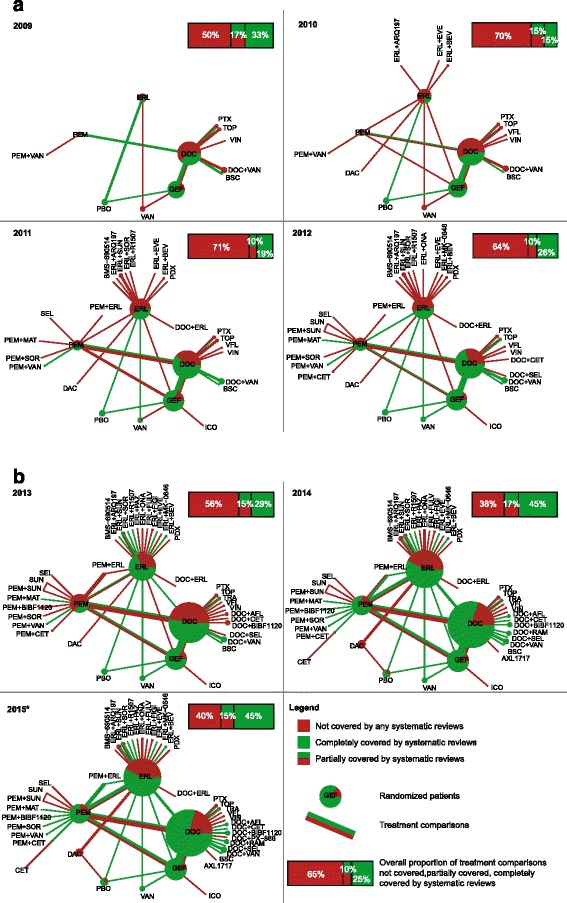
Cumulative networks of evidence showing the gap between the amount of randomized evidence covered by systematic reviews and the amount of randomized evidence available for inclusion. (**a**) 2009–2012 and (**b**) 2013–2015. ^*^The last search for randomized trials and systematic reviews was conducted on March 2, 2015. From 2009 to 2015, we compared randomized controlled trials selected by systematic reviews published up to December 31 each year (up to March 2 for 2015) to all trials eligible for inclusion (i.e., all trial results published up to July 1 each year [up to August 31, 2014 for 2015]). Each node size is proportional to the total number of patients randomly allocated to the corresponding treatment across all randomized trials available for inclusion; we represented the proportion of randomized patients actually covered by systematic reviews by pie charts overlaid on nodes in the network. The thickness of each edge is proportional to the total number of randomized controlled trials between the corresponding treatments available for inclusion; we represented the proportion of trials actually selected by systematic reviews by a percentage bar chart overlaid on edges in the network

The cumulative networks of evidence show how the mismatch between available data and syntheses persisted from 2009 to 2015 (Fig. 4). Across all years, 10 % to 17 % of treatment comparisons were partially covered by systematic reviews and 55 % and 85 % were partially or not covered by systematic reviews. Moreover, the proportion of evidence covered by systematic reviews was unequally distributed across treatments.

Sensitivity analyses gave consistent findings. When removing trials of drugs that did not pass phase II, 27 % to 64 % of trials, 27 % to 58 % of patients, 22 % to 53 % of treatments, and 21 % to 67 % of treatment comparisons were missing. When removing trials that did not report overall survival or progression-free survival, 41 % to 68 % of trials, 29 % to 58 % of patients, 36 % to 63 % of treatments, and 36 % to 69 % of treatment comparisons were missing. When discarding trials reported in conference abstracts only, 37 % to 68 % of trials, 24 % to 58 % of patients, 33 % to 63 % of treatments, and 33 % to 68 % of treatment comparisons were missing. When the lag period was the last date of search of the last systematic review, 42 % to 70 % of trials, 11 % to 53 % of patients, 36 % to 66 % of treatments, and 37 % to 72 % of treatment comparisons were missing.

## Discussion

In this study, we assessed whether the whole set of conventional meta-analyses on pairwise treatment comparisons would allow for meeting clinicians’ and patients’ needs, to provide a comprehensive, up-to-date synthesis of evidence for all treatments. Our comparison of the amount of randomized evidence covered by systematic reviews and all randomized trials available for inclusion revealed a substantial waste related to the failure of systematic reviews to accumulate evidence scientifically: the evidence covered by existing systematic reviews on the topic was always substantially incomplete, with 40 % or more of treatments, treatment comparisons, and trials missing.

All meta-analyses on the same topic evaluating only a small fragment of the evidence has consequences for patient care and research planning. We need to identify the treatments with harmful effects and also treatments with side effects that have no advantage as compared with alternative treatments. As well, we need to be able to identify the treatment or group of treatments that works best. The relative beneficial effect of a treatment may be missed if some treatment comparisons of interest are not covered by systematic reviews. In our example, no systematic review encompassed all available treatments. Moreover, encompassing all the evidence for all treatments may have important implications for planning subsequent trials and helping prioritize future research to improve the evidence base [46]. In fact, based on the synthesis results and the geometry of the network of evidence, one could design a trial of treatments infrequently compared or a trial of the best potential treatment [47, 48].

Several reasons explain why the 29 systematic reviews did not cover all the randomized evidence. First, the trials may have addressed narrow and focused questions as compared to each review’s selection criteria (patients, interventions, and comparators). However, all trials we identified would have been eligible for inclusion in at least one systematic review; missing trials were not excluded from systematic reviews because they were not eligible.

Second, many meta-analyses become quickly out-of-date, sometimes by the time they are published. In fact, the systematic identification of trials is complex and time-consuming. In our case study, only one of 29 reviews was an update of a previous review and a large proportion of treatment comparisons covered by systematic reviews was out-of-date. This updating issue may be improved by the automation, as much as possible, of the whole trial search and selection process. These automated technologies would alleviate the burden on systematic reviewers. Third, another reason for the failure of systematic reviews to accumulate all available evidence is inadequate search methods for unpublished trials. In our case study, among the 15 trials (20 %) with unpublished results, only three (20 %) were included in systematic reviews. Among the 29 systematic reviews, 79 % could be considered at high risk of missing trials that would have met the inclusion criteria because they did not report duplicate study selection and data extraction, comprehensive literature search, or searching for reports regardless of publication type. Automated processes, such as meta-search engines, could systematically cover sources such as industry and non-industry trial registries and results databases to identify posted results and improve the evidence synthesis [49, 50]. Beyond the example at hand, the current way of conducting systematic reviews explains why they are inherently at risk of providing a fragmented, out-of-date panorama of the evidence for all treatments.

A broader scope on all evidence available for all treatments of a given condition is naturally provided by a systematic review with network meta-analysis, which allows for examining the totality of the randomized evidence using trial networks [51, 52]. Although the production of network meta-analyses follows an exponential growth, their number is still relatively limited [53]. With the increasing development of new treatments, examining networks of randomized trials is essential, and multiple treatment comparisons cannot be avoided [54]. Conventional meta-analysis focuses on comparisons of two treatments only. However, network meta-analysis allows for comparing all treatments to each other with direct and indirect comparisons, even if randomized trials are not available for some treatment comparisons. In our example, the available direct evidence informed 5 % of all possible comparisons between the 47 treatments. Ideally, the scoping exercise to define the criteria for considering trials in the systematic review should include all alternative treatments for the target condition [55]. It is still possible that a network meta-analysis will selectively choose treatments to include in the network, and excluding treatments may affect estimated treatment effects [56]. In our study, we identified two network meta-analyses; neither covered the whole evidence available at their time of analysis and considered a very restrictive network instead. Finally, network meta-analysis offers more flexibility to assess individual treatments. It offers the opportunity to overcome a common issue in standard meta-analysis whereby different treatments and sometimes different types of treatments are lumped together. In our example, erlotinib and gefitinib were frequently lumped together in EGFR tyrosine kinase inhibitors and docetaxel and pemetrexed were lumped together in monochemotherapy. This type of evidence lumping does not provide the relevant information for clinicians and patients who want to know which specific treatments work the best.

The paradox is that the set of systematic reviews fail to cover all the evidence, diminishing the value of research to clinicians by missing important comparisons. In fact, the production of meta-analyses has been substantially and rapidly increasing worldwide [57, 58]. In parallel, the evidence covered by systematic reviews features many gaps, and multiple overlapping meta-analyses on the same topic are common [59, 60].

**Fig. 5:**
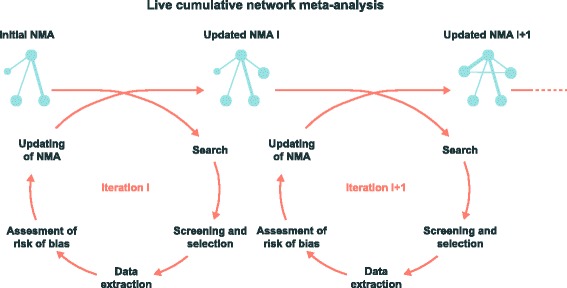
A new approach to synthesize evidence: live cumulative network meta-analysis. Starting from an initial NMA, a research community would regularly (e.g., every 3 months), search for, screen, and select trials with new results and, if any, extract data, assess the risk of bias, and update the NMA. NMA: network meta-analysis

As an alternative to classical systematic reviews performed at one point in time, a new theoretical framework is “living systematic reviews”, defined as high-quality online summaries of health research updated as new research becomes available [61]. We propose to push further the shift towards a new paradigm by switching: 1) from a series of standard meta-analyses focused on specific treatments (many treatments being not considered) to a single network meta-analysis covering all treatments; and 2) from meta-analyses performed at a given time and frequently out-of-date to a cumulative network meta-analysis systematically updated as soon as the results of a new trial become available, an approach to synthesis we call “live cumulative network meta-analysis”. In Fig. 5, we show the methodological steps we propose for live cumulative network meta-analysis.

We acknowledge that developing such methodology is challenging. In Table 2, we present some key challenges and potential solutions. The rigorous methodology of systematic reviews (exhaustive search of trials, minimizing subjectivity by independent duplicate assessments, assessing risk of bias within trials) is inherently demanding of resources and time, especially for a systematic review incorporating network meta-analysis. Moreover, keeping a systematic review up-to-date requires processes closer to those of rapid reviews (i.e., using accelerated and streamlined methods). Therefore, there is necessarily a trade-off between high-standard synthesis methods and real-time updating processes. Automated technologies may help define this trade-off by alleviating the burden of manual tasks for systematic reviewers. Several tools have been proposed to improve, hasten, and ease the search for trials, trial selection, extraction of data, and assessment of risk of bias [49, 50, 62, 63]. Live cumulative network meta-analysis may also raise issues regarding the current authoring and publishing system. Online posting may be more adequate to report periodically the findings of such “real-time” syntheses. Since Elliott et al. presented the theoretical framework of living systematic reviews, some examples have been published and have only partly addressed the aforementioned challenges [64–68]; for instance, by using accompanying open-access websites to disseminate the updates of the systematic review.

**Table 2:** Methodological steps of live cumulative network meta-analysis, key challenges, and potential solutions

Methodological steps	Key challenges	Potential solutions
0. Initial network meta-analysis	Resource intensive but commonly one-shot investment	Setting-up of a research community (preferentially international) in charge of designing a high-quality and clinically relevant network meta-analysis and keeping it up-to-date for a given mandate (e.g., a 5- or 10-year period)
	Redundant meta-analyses frequently commissioned by different groups	
	Need to consider all patient-important outcomes	
Perform iterations at regular intervals (e.g., every 3 months) through steps 1–5		
1. Search for trials	Need to identify trials of novel drugs. For instance, six to nine new second-line therapies per year in advanced NSCLC	Community expert monitoring would identify pipeline therapies assessed in clinical trials and allow adapting the search equations
	Querying repeatedly a wide range of sources to identify trials with published and unpublished results is time consuming and labor intensive	Metasearch engine script designed for the question at hand would allow querying automatically and simultaneously the multiple sources [75]
	Need to identify multiple reports of the same trial. For instance, there were on average two reports per trial of second-line treatments in advanced NSCLC	The OpenTrials database would contain all openly available data and documents on all clinical trials threaded together by trial ID [76]
	Need to update the list of treatments, of trials, and multiple reports for the same trial	
2. Screening of reports and selection of trials	Screening repeatedly may be resource intensive depending on the clinical question. In second-line therapies of advanced NSCLC we estimated that the workload would be manageable (about 50 new records to screen each month for CENTRAL, MEDLINE, EMBASE, and around 600 conference abstracts per year)	Using crowdsourcing for screening would allow distributing microtasks to community experts and dealing with increasing amounts of evidence [77, 78]
		Future automated technologies would help community experts in the screening process; for instance, natural language processing methods using the semantic features of the reports and could help identify potentially relevant trial reports [49, 50, 79–82]
If required only (at least one trial with new results), continue with steps 3–5		
3. Data extraction	Extracting data and assessing the risk of bias repeatedly may be resource intensive depending on the number of trials with new results. In second-line therapies of advanced NSCLC we estimated that the workload would be manageable (about 10 to 15 new trials per year)	Using crowdsourcing for data extraction would allow distributing microtasks to experts and dealing with increasing amounts of evidence [77, 83]
4. Assessment of risk of bias		
	Need to check for consistency in extracted data between multiple reports for the same trial; in cases of inconsistency, need to justify the choice of a specific source	Automatic data extraction is possible depending on the source. For instance, it is possible to abstract automatically posted results from ClinicalTrials.gov [84–86]
		Future automated technologies could help experts to extract data or to assess the risk of bias within trials [49, 50, 62, 63]
5. Updating of network meta-analysis	Need to develop online software for updating the network meta-analysis^*^	Online solutions in development for conventional meta-analysis could be extended to network meta-analysis [87, 88]
6. Dissemination	Need to make the results publicly available after each iteration	A freely accessible website would allow reporting the live cumulative network meta-analysis, including all details regarding methods and processes, graphs, and data
	Need for transparent reporting of the whole process	
	Need for peer-review	Alternative forms of peer-review (e.g., post-publication peer-review) could be implemented

^*^Eventually incorporating adjustment for multiple testing in live cumulative network meta-analysis to account for the inflated type I error, depending on ongoing discussion [89]. NSCLC: non-small cell lung cancer

Another challenge would be to consider all outcomes that are important or critical to patients for decision making in these live cumulative network meta-analyses [69, 70]. In our case study, we included trials regardless of reported outcomes; in a sensitivity analysis, we excluded trials that did not report treatment effects on overall survival or progression-free survival. However, there are other patient-important outcomes, in particular to measure the symptom burden of the disease and the quality of life of patients. More generally, it will be crucial to consider networks of trials according to the reporting of the different patient-important outcomes. In fact, the geometry of the network of trials could vary across outcomes because of differential reporting of outcomes (e.g., efficacy and safety outcomes) across drugs and trials.

Nonetheless, embracing the perspective of networks of trials of all alternative treatments for each condition, and in particular developing live cumulative network meta-analyses, could greatly benefit various stakeholders, including physicians, patients, and also guideline developers, funders, and decision-makers [71]. Networks of trials and their synthesis through network meta-analysis could increase the value of research when treatment recommendations are based on an exhaustive up-to-date network of randomized evidence [72]. Guideline developers and other decision-makers may further benefit from network meta-analyses if these implement recent developments to rate the quality of the body of evidence supporting treatment effect estimates for all patient-important outcomes (e.g. GRADE Summary of Findings tables) and rankings from network meta-analysis. For instance, in the field of medical therapies for open angle glaucoma, Li et al. recently showed that if a network meta-analysis had been conducted earlier, prostaglandins could have been shown as the most effective class in lowering intraocular pressure 7 years ahead of the guideline recommendation [73]. Moreover, biomedical research funding agencies could improve the prioritization of research proposals if they had access to a mapping of existing (and ongoing) trials evaluating all available treatments for a specific condition at the time a new trial is planned. Visualizing the network of trials and identifying which new trial maximizes the information can help stakeholders assess gaps in evidence and choose the next treatment comparison or trial that needs to be prioritized.

To our best knowledge, our study is the first to highlight the substantial waste associated with the failure of systematic reviews to accumulate evidence across all treatments (for the same disease). Moreover, our methodology based on a series of systematic overviews and networks of randomized evidence is novel and could be replicated in other fields. Our study has several limitations. First, we examined only one topic, second-line treatments for advanced NSCLC. However, our results should be generalizable, because the scope of meta-analyses is frequently narrow, with 81 % of standard meta-analyses that do not include all treatments and 43 % that cover only specific treatments [7]. Contrary to Haidich and colleagues, who assessed the evidence at the level of each systematic review, we assessed the cumulative evidence covered by all systematic reviews on a topic with a “bird’s-eye view”. Second, our gold standard included trials that meta-analysts may have considered ineligible for inclusion in their systematic reviews. However, we performed several sensitivity analyses and results were consistent. Third, we excluded trials in which the control group received different chemotherapy drugs at the discretion of the investigators. Such trials could also contribute to the synthesis in a network meta-analysis with a class effect model [74]; however, they do not allow for assessing the relative merits of the specific drugs. Similarly, we excluded randomized trials and systematic reviews comparing two different administration schemes because our focus was the comparison of alternative treatments against each other. Nonetheless, our analysis could be extended to such randomized trials and systematic reviews. In such cases, each relevant node would have several subnodes that relate to different administration schemes. Fourth, we may have missed some systematic reviews and randomized trials because of reporting bias, but we tried to minimize this possibility by an exhaustive search covering conference abstracts and registries. Fifth, we started our analysis for the year 2009; this pre-specified year was somehow arbitrary and we acknowledge that this starting point could have been earlier, considering that the first systematic review was published in April 2001; the second one was published in December 2005. However, there was no or little randomized evidence available between competing treatments at that time. Finally, we did not perform any outcome data synthesis at this stage. One may ask if covering all the randomized evidence available would have led to clinically important differences for the 10 % to 17 % of treatment comparisons partially covered by systematic reviews. In the framework we are promoting, a network meta-analysis would allow for estimating all treatment comparisons. Adding up to 40 % of missing evidence (about 8,000 patients) to the network would likely lead to clinically important differences, in particular for treatment comparisons partially covered by systematic reviews.

## Conclusions

We illustrated that systematic reviews of a given condition provide a fragmented, out-of-date panorama of the evidence for all treatments. Embracing the perspective of networks of trials of all alternative treatments for each condition can have important consequences and should be adopted more generally. The waste of research associated with the failure to accumulate evidence could be reduced by the development of live cumulative network meta-analyses.

## Additional file

Additional file 1:
**Details regarding the methods and results.** Appendix 1. List of eligible treatments. Appendix 2. Search equations for randomized controlled trials. Appendix 3. Other sources searched to identify randomized controlled trials. Appendix 4. Search equations for systematic reviews. Appendix 5. Other sources searched to identify systematic reviews. Appendix 6. Cumulative number of systematic reviews of second-line treatments in advsanced non-small cell lung cancer from 2009 to March 2015. (DOC 223 kb)

## Abbreviations

ALK: anaplastic lymphoma kinase
EGFR: epidermal growth factor receptor
NSCLC: non-small cell lung cancer
