# Supplementary material for: Wasted research when systematic reviews fail to provide a complete and up-to-date evidence synthesis: the example of lung cancer
Source: BMC Med. 2016 Jan 20;14:8. doi: 10.1186/s12916-016-0555-0 (PMC4719540; doi:10.1186/s12916-016-0555-0)
Supplement: Additional file 1: — Details regarding the methods and results. Appendix 1. List of eligible treatments. Appendix 2. Search equations for randomized controlled trials. Appendix 3. Other sources searched to identify randomized controlled trials. Appendix 4. Search equations for systematic reviews. Appendix 5. Other sources searched to identify systematic reviews. Appendix 6. Cumulative number of systematic reviews of second-line treatments in advsanced non-small cell lung cancer from 2009 to March 2015. (DOC 223 kb) [file 12916_2016_555_MOESM1_ESM.doc]

Appendix

Appendix 1: List of eligible treatments

| Monochemotherapy | Docetaxel, Ifosfamide, Irinotecan, Gemcitabine, Mitomycin, Paclitaxel, Pemetrexed, Vinblastine, Vindesine, Vinorelbine |
| --- | --- |
| Targeted treatments | Afatinib, Bevacizumab, Cetuximab, Erlotinib, Gefitinib, Icotinib, Nintedanib, Ramucirumab, Sorafenib, Sunitinib, Vandetanib |

Appendix 2: Search equations for randomized controlled trials*

MEDLINE

| Patients |  |  |
| --- | --- | --- |
| Lung cancer | #1 | lung neoplasms[MeSH] OR carcinoma, non small cell lung[MeSH] OR lung carcinom*[tiab] OR lung neoplasm*[tiab] OR lung cancer[tiab] OR NSCLC[tiab] OR non small cell lung[tiab] |
| Advanced | #2 | "Neoplasm Metastasis"[MeSH] OR advanced[tiab] OR stage IV[tiab] OR stage 4[tiab] OR stage four[tiab] OR metastatic[tiab] OR metastases[tiab] |
| Intervention |  |  |
| Chemotherapy | #3 | chemotherapy[tiab] OR chemotherapies[tiab] OR docetaxel[tiab] OR taxotere[tiab] OR "docetaxel"[Supplementary Concept] OR pemetrexed[tiab] OR alimta[tiab] OR "pemetrexed"[Supplementary Concept] OR gemcitabine[tiab] OR "gemcitabine"[Supplementary Concept] OR vinorelbine[tiab] OR "vinorelbine"[Supplementary Concept] OR paclitaxel[tiab] OR "Paclitaxel"[MeSH] OR "Vinblastine"[Supplementary Concept] OR vinblastine[tiab] OR "Ifosfamide"[MeSH] OR Ifosfamide[tiab] OR "irinotecan"[Supplementary Concept] OR irinotecan[tiab] OR "Mitomycin"[MeSH] OR mitomycin[tiab] OR vindesine[tiab] OR "Vindesine"[MeSH] |
| EGFR Targeted therapy | #4 | "protein kinase inhibitors"[MeSH] OR "kinase inhibitor"[tiab] OR "kinase inhibitors"[tiab] OR gefitinib[tiab] OR iressa[tiab] OR "gefitinib"[Supplementary Concept] OR erlotinib[tiab] OR tarceva[tiab] OR "erlotinib"[Supplementary Concept] OR "icotinib"[Supplementary Concept] OR "icotinib"[tiab] OR afatinib[tiab] OR "BIBW 2992" [Supplementary Concept] OR "cetuximab"[Supplementary Concept] OR cetuximab[tiab] |
| VEGF targeted therapy | #5 | bevacizumab[tiab] OR "bevacizumab"[Supplementary Concept] OR "vandetanib"[tiab] OR sunitinib[tiab] OR "sunitinib"[Supplementary Concept] OR sorafenib[tiab] OR "sorafenib"[Supplementary Concept] OR Ramucirumab[tiab] OR "ramucirumab"[Supplementary Concept] OR "nintedanib"[Supplementary Concept] OR nintedanib[tiab] |
| Second line | #6 | second line[tiab] OR pretreat*[tiab] OR previously treated[tiab] OR refractory[tiab] OR recurrent[tiab] |
| Cochrane Highly Sensitive Search Strategy | #7 | (randomized controlled trial[Publication Type] OR controlled clinical trial[Publication Type] OR randomized[tiab] OR placebo[tiab] OR drug therapy[sh] OR randomly[tiab] OR trial[tiab] OR groups[tiab]) NOT (animals[mh] NOT humans[mh]) |
| Search equation | #8 | #1 AND #2 AND (#3 OR #4 OR #5) AND #6 AND #7 |

EMBASE

| Patients |  |  |
| --- | --- | --- |
| Lung cancer | #1 | 'lung tumor'/exp OR 'non small cell lung cancer'/exp OR (lung NEXT/1 carcinom*):ab,ti OR (lung NEXT/1 neoplasm*):ab,ti OR 'lung cancer':ab,ti OR nsclc:ab,ti OR 'non small cell lung':ab,ti |
| Advanced | #2 | 'metastasis'/exp OR advanced:ab,ti OR 'stage IV':ab,ti OR 'stage 4':ab,ti OR 'stage four':ab,ti OR metastatic:ab,ti OR metastases:ab,ti |
| Intervention |  |  |
| Chemotherapy | #3 | 'chemotherapy':ab,ti OR 'chemotherapies':ab,ti OR docetaxel:ab,ti OR taxotere:ab,ti OR 'docetaxel'/exp OR pemetrexed:ab,ti OR alimta:ab,ti OR 'pemetrexed'/exp OR gemcitabine:ab,ti OR 'gemcitabine'/exp OR vinorelbine:ab,ti OR 'navelbine'/exp OR paclitaxel:ab,ti OR 'paclitaxel'/exp OR 'vinblastine'/exp OR vinblastine:ab,ti OR 'ifosfamide'/exp OR ifosfamide:ab,ti OR 'irinotecan'/exp OR irinotecan:ab,ti OR 'mitomycin'/exp OR mitomycin:ab,ti OR vindesine:ab,ti OR 'vindesine'/exp |
| EGFR Targeted therapy | #4 | 'protein kinase inhibitor'/exp OR 'kinase inhibitors':ab,ti OR 'kinase inhibitor':ab,ti OR gefitinib:ab,ti OR iressa:ab,ti OR 'gefitinib'/exp OR erlotinib:ab,ti OR tarceva:ab,ti OR 'erlotinib'/exp OR 'icotinib'/exp OR icotinib:ab,ti OR afatinib:ab,ti OR 'afatinib'/exp OR 'bibw 2992':ab,ti OR 'cetuximab'/exp OR cetuximab:ab,ti |
| VEGF targeted therapy | #5 | bevacizumab:ab,ti OR 'bevacizumab'/exp OR vandetanib:ab,ti OR 'vandetanib'/exp OR sunitinib:ab,ti OR 'sunitinib'/exp OR sorafenib:ab,ti OR 'sorafenib'/exp OR ramucirumab:ab,ti OR 'ramucirumab'/exp OR 'nintedanib'/exp OR nintedanib:ab,ti |
| Second line | #6 | 'second line':ab,ti OR pretreat*:ab,ti OR 'previously treated':ab,ti OR 'refractory':ab,ti OR 'recurrent':ab,ti |
| Filter used by the UK Cochrane Centre | #7 | 'crossover procedure'/exp OR 'double-blind procedure'/exp OR 'randomized controlled trial'/exp OR 'single-blind procedure'/exp OR random* OR factorial* OR crossover* OR cross NEXT/1 over* OR placebo* OR doubl* NEAR/1 blind* OR singl* NEAR/1 blind* OR assign* OR allocat* OR volunteer* |
| Search equation | #8 | #1 AND #2 AND (#3 OR #4 OR #5) AND #6 AND #7 |

## Appendix 3: Other sources searched to identify randomized controlled trials

3.1. List of pharmaceutical companies and corresponding drugs

| Pharmaceutical company | Drugs | Clinical trial results database |
| --- | --- | --- |
| AstraZeneca | Fulvestrant, Gefitinib, Selumetinib | <http://www.astrazenecaclinicaltrials.com/> |
| Bayer | Sorafenib | http://pharma.bayer.com/en/research-and-development/clinical-trials/trial-finder/index.php |
| Boehringer Ingelheim | Nintedanib,  Afatinib | <http://trials.boehringer-ingelheim.com/trial_results/clinical_trials_overview/clinical_trial_result.c=i.i=10.html> |
| GlaxoSmithKline | Pazopanib, Topotecan, Trametinib, Vinorelbine | <http://www.gsk-clinicalstudyregister.com/> |
| Lilly | Pemetrexed,  Gemzar | <http://lillytrials.com/results/alimta.pdf?bcsi_scan_628cd39dca2568d2=0&bcsi_scan_filename=alimta.pdf>  http://lillytrials.com/results/Gemzar.pdf?bcsi_scan_628cd39dca2568d2=0&bcsi_scan_filename=Gemzar.pdf |
| Novartis | Everolimus | <http://www.novctrd.com/ctrdWebApp/clinicaltrialrepository/public/login.jsp> |
| Roche | Bevacizumab, R1507, Tarceva, Onartuzumab | <http://www.roche-trials.com/searchFullText.action?drug=2> |
| Sanofi | Aflibercept, Docetaxel | <http://en.sanofi.com/rd/clinical_trials/our_commitments/clinical_study_results.aspx> |

3.2. Regulatory agency online databases

- Food and Drug Administration

<http://www.accessdata.fda.gov/scripts/cder/drugsatfda/>

Drugs: Docetaxel, Fulvestrant, Gefitinib, Irinotecan, Paclitaxel, Pemetrexed ,Vinorelbine

- European Medicines Agency <http://www.ema.europa.eu/ema/index.jsp?curl=pages%2Fmedicines%2Flanding%2Fepar_search.jsp&mid=WC0b01ac058001d125&searchTab=&alreadyLoaded=true&isNewQuery=true&status=Authorised&status=Withdrawn&status=Suspended&status=Refused&keyword=Enter+keywords&searchType=name&taxonomyPath=Diseases.Cancer.Neoplasms+by+Site.Thoracic+Neoplasms.Respiratory+Tract+Neoplasms.Lung+Neoplasms.Carcinoma%2C+Bronchogenic&treeNumber=&currentCategory=Carcinoma%2C+Non-Small-Cell+Lung&searchGenericType=generics>

Drugs: Afatinib, Bevacizumab, Docetaxel, Erlotinib, Gefitinib, Nintedanib, Paclitaxel, Pemetrexed, Topotecan

3.3. Conference abstracts

- American Society of Clinical Oncology Meeting from 2009 to 2014:
- 2009: <http://meeting.ascopubs.org/content/vol27/15_suppl> and <http://meeting.ascopubs.org/content/vol27/18_suppl>
- 2010: <http://meeting.ascopubs.org/content/vol28/15_suppl> and <http://meeting.ascopubs.org/content/vol28/18_suppl>
- 2011: <http://meeting.ascopubs.org/content/vol29/15_suppl> and http://meeting.ascopubs.org/content/vol29/18_suppl
- 2012 to 2014: <http://meeting.ascopubs.org/>

Section: [Lung Cancer - Non-small Cell Metastatic](http://meeting.ascopubs.org/search?tocsectionid=Lung+Cancer-Non-small+Cell+Metastatic&displaysectionid=Lung+Cancer-Non-small+Cell+Metastatic&volume=31&issue=18_suppl&hits=10&submit=Submit)

- European Society of Medical Oncology Congress from 2009 to 2014:
- European Journal of Cancer Supplements, Volume 7, issue 2, September 2009
- Annals of Oncology 21 (Supplement 8): viii122–viii161, 2010. Section non-small cell lung cancer, metastatic
- European Journal of Cancer, Volume 47 Supplement 1, September 2011
- Annals of Oncology 23 (Supplement 9): ix400–ix446, 2012. Section non-small cell lung cancer, metastatic
- European Journal of Cancer, Volume 49 Supplement 2, September 2013. Section Lung Cancer – Metastatic
- Annals of Oncology 25 (Supplement 4): iv426–iv470, 2014. Section non-small cell lung cancer, metastatic
- World Lung Cancer Conference from 2009 to 2014

2009: Journal of Thoracic Oncology, volume 4, number 9, supplement 1, September 2009. Section: NSCLC - Advanced Disease

2011: <http://abstracts.webges.com/wclc2011/myitinerary>

Section: NSCLC - Advanced Stage

2013:<http://abstracts.webges.com/wclc2013/myitinerary>

Keyword: Non-small cell lung cancer. Section: Medical Oncology

Appendix 4: Search equations for systematic reviews

MEDLINE

| Patients |  |  |
| --- | --- | --- |
| Lung cancer | #1 | lung neoplasms[MeSH] OR carcinoma, non small cell lung[MeSH] OR lung carcinom*[tiab] OR lung neoplasm*[tiab] OR lung cancer[tiab] OR NSCLC[tiab] OR non small cell lung[tiab] |
| Intervention |  |  |
| Second line | #2 | second line[tiab] OR pretreat*[tiab] OR previously treated[tiab] OR refractory[tiab] OR recurrent[tiab] |
| Methods filter |  |  |
| health-evidence.ca systematic review methodology filter | #3 | MEDLINE[tw] or systematic review[ tw] or meta-analysis [pt] or intervention$[ti] |
| Pubmed systematic reviews subset strategy | #4 | systematic [sb] |
| Search equation | #5 | #1 AND #2 AND (#3 OR #4) |

EMBASE

| Patients |  |  |
| --- | --- | --- |
| Lung cancer | #1 | 'lung tumor'/exp OR 'non small cell lung cancer'/exp OR (lung NEXT/1 carcinom*):ab,ti OR (lung NEXT/1 neoplasm*):ab,ti OR 'lung cancer':ab,ti OR nsclc:ab,ti OR 'non small cell lung':ab,ti |
| Intervention |  |  |
| Second line | #2 | 'second line':ab,ti OR pretreat*:ab,ti OR 'previously treated':ab,ti OR 'refractory':ab,ti OR 'recurrent':ab,ti |
| health-evidence.ca Systematic review filter | #3 | medline:ab,ti OR 'systematic review'/exp OR 'systematic review':ab,ti OR 'meta analysis'/exp OR intervention*:ti |
|  | #4 | #1 AND #2 AND #3 |
|  | #5 | #4 AND [embase]/lim |
|  | #6 | #4 AND [medline]/lim |
| Search equation | #7 | #5 NOT #6 |

## Appendix 5: Others sources searched to identify systematic reviews

5.1. Conference abstracts

As previously described in Appendix 3, section 3.3.

Keywords:

- non-small cell lung cancer, systematic reviews

- non-small cell lung cancer, meta-analysis

5.2. Prospero Register of systematic reviews

<http://www.crd.york.ac.uk/PROSPERO/>

Keywords: non-small cell lung cancer, second line

Review status : completed

Appendix 6: Cumulative number of systematic reviews of second-line treatments in advanced non-small cell lung cancer from 2009 to March 2015.

* The last search for randomized controlled trials and systematic reviews was conducted on March 2, 2015.
